# Seed management using NGS technology to rapidly eliminate a deleterious allele from rice breeder seeds

**DOI:** 10.1270/jsbbs.22058

**Published:** 2022-12-13

**Authors:** Elias G. Balimponya, Maria S. Dwiyanti, Toshiaki Ito, Shuntaro Sakaguchi, Koichi Yamamori, Yoshitaka Kanaoka, Yohei Koide, Yoshifumi Nagayoshi, Yuji Kishima

**Affiliations:** 1 Research Faculty of Agriculture, Hokkaido University, Sapporo, Hokkaido 060‑8589, Japan; 2 Miyazaki Comprehensive Agricultural Experiment Station, Miyazaki 880-0212, Japan

**Keywords:** albino, breeder seeds, next-generation sequencing (NGS), rice, seed management

## Abstract

Spontaneous mutations are stochastic phenomena that occur in every population. However, deleterious mutated allele present in seeds distributed to farmers must be detected and removed. Here, we eliminated undesirable mutations from the parent population in one generation through a strategy based on next-generation sequencing (NGS). This study dealt with a spontaneous albino mutant in the ‘Hinohikari’ rice variety grown at the Miyazaki Comprehensive Agricultural Experiment Station, Japan. The incidence of albinism in the population was 1.36%. NGS analysis revealed the genomic basis for differences between green and albino phenotypes. Every albino plant had a C insertion in the *Snow-White Leaf1* (*SWL1*) gene on chromosome 4 causing a frameshift mutation. Selfing plants heterozygous for the mutant allele, *swl1-R332P*, resulted in a 3:1 green/albino ratio, confirming that a single recessive gene controls albinism. Ultrastructural leaf features in the *swl1-R332P* mutants displayed deformed chlorophyll-associated organelles in albino plants that were similar to those of previously described *swl1* mutants. Detection of the causative gene and its confirmation using heterozygous progenies were completed within a year. The NGS technique outlined here facilitates rapid identification of spontaneous mutations that can occur in breeder seeds.

## Introduction

Mutations occur at an extremely low but constant rate of approximately 10^–9^/nucleotides/cell division (Kinde *et al.* 2011, Milholland *et al.* 2017), and this steady source of variability acts as a driving force in evolution. Indeed, the source of biological diversity owes much to mutation as well as recombination (Clarke 1975). Crop breeding also relies on harnessing certain mutations that suit a particular purpose. Natural mutations provide an abundant resource for living organisms.

Nonetheless, in crop breeding, once a variety has been optimized, the occurrence of any additional mutations is unwelcome because it undercuts the crucial requirements of Distinctness, Uniformity, and Stability (DUS) for crop varieties (Jones *et al.* 2003). Mutations are the driving force for genetic variation in organisms (Nei 2007), but outcrossing, volunteer plants, and admixture can also perturb DUS within cultivars (Parlevliet 2007). The occurrence of mutations within a variety may disturb its DUS and subsequently prevent it from being a useful breeding population. However, since mutations occur equally in all organisms (Linnane *et al.* 1989), even a completely homozygous variety is not immune to genetic mutation. Therefore, it is extremely important for breeders to control the accumulation of genetic mutations and prevent harmful mutations from spreading. However, many mutations that occur in the genome of a crop plant do not affect desirable traits and are therefore difficult to detect (Slade and Knauf 2005). In addition, mutations take several generations to spread through the population to become sufficiently frequent for detection (Distante *et al.* 2004). Therefore, it is likely that even harmful mutations are often overlooked. Moreover, it is difficult or impossible to visually detect individuals with mutant genes when they are heterozygous (Yang *et al.* 2015). Mutations thus pose a threat to the management of a variety, since the appearance of a harmful trait will decrease its use and value.

Advances in next-generation sequencing (NGS) technology have facilitated gene identification and the elucidation of detailed genome structures (Ku *et al.* 2011), while also enabling the detection of single-nucleotide polymorphisms (SNPs) and small insertion-deletion mutations (indels) in genome-wide comparative analysis (Grossmann *et al.* 2011). Additionally, NGS has become widely used in the evaluation and classification of genetic resources (Abdelrahman *et al.* 2018, Machida-Hirano and Niino 2017, Nybom and Lācis 2021, Wambugu *et al.* 2018). However, the application of NGS technology to crop variety management, especially for quality control of breeder seeds and other products, is still in its infancy. Depending on the cost-effectiveness (Stoddard *et al.* 2014, Torchia *et al.* 2019), NGS technology can be extremely useful for strict adherence to the DUS of a variety, facilitating the detection of genomic variation within a population and of potentially serious mutations, and thereby limiting their harmful effects.

In this study, we analyzed rice (*Oryza sativa*) breeder seeds that occasionally produce albino mutant individuals to identify the causal genes and develop a way to detect them even in heterozygotes. The causative gene, *Snow-White Leaf 1* (*SWL1*) (Hayashi-Tsugane *et al.* 2014), was found to be frameshifted by a single-nucleotide insertion. The analyses leading to this detection were performed in less than a year using NGS on two bulk samples, albino and wild type, which showed that the albino phenotype was closely related to the mutation in *SWL1*. Chloroplast structure in the observed *swl1-R332P* mutants resembled that observed during the initial discovery of the *SWL1* gene, where the thylakoid membrane was not visible in *swl1* mutant plants. Moreover, the same transcript (Os04g0497900) of *SWL1* was affected, although the previously described mutation consisted of a transposon inserted into the 5ʹ-untranslated region leading to a variegated leaf phenotype (Hayashi-Tsugane *et al.* 2014), whereas our investigation uncovered a C insertion in the coding region of the gene that leads to complete albinism. We were also able to identify individuals with heterozygous genotypes.

Conventional forward-genetics methods using map-based cloning (Peters *et al.* 2003, Saito *et al.* 2010) require several years for generation cycling, marker creation, and cloning. In comparison, the NGS-based method in this study was extremely effective in identifying and removing harmful genes latent in the breeder seed population in approximately 1 year. Regarding the identification of *swl1-R332P*, and the association between this mutation and the albinism phenotype, we compared the intracellular ultrastructure of previously reported *swl1* mutants with those of our own albino individuals and observed marked similarities between them. We propose a seed management method that employs NGS to remove harmful genes from the breeder seed population to minimize the accumulation of deleterious mutations.

## Materials and Methods

### Experimental materials

The albino mutants were found in breeder seeds for the ‘Hinohikari’ variety of *O. sativa* spp. *japonica*. The ‘Hinohikari’ variety was developed as an F_6_ pedigree progeny of ‘Koganebare’/‘Koshihikari’ at the Miyazaki Comprehensive Agricultural Experiment Station, Japan (MCAES) in 1979 (Yagi *et al.* 1990). The presence of albinism in the breeder seeds was discovered in 2015, and albino plants currently arise in the population at a consistent frequency of 1.36% (Table 1). One week after sowing in soil with compost, rice seedlings grown in a greenhouse at 25°C clearly included both albino and green plants.

### DNA isolation from albino and green plants for pool samples for NGS

Rice seedlings were sampled 6 weeks after planting, and the entire aboveground portion of the plants was used for DNA isolation. The DNeasy^®^ Plant Mini Kit extraction protocol (Qiagen) was used following the manufacturer’s instructions with small modifications. For DNA pools, the amount of DNA from each sample in the pool was set at 10 ng, and 10 and 13 samples of albino and green plants, respectively (Supplemental Table 1), were pooled together in each group. Before pooling, the DNA quality and quantity were checked with a NanoDrop spectrophotometer and gel electrophoresis with 1% agarose gel in 0.5× TBE (Tris-Borate-EDTA).

### Next-generation sequencing and analysis

The gDNA of pooled samples was sent to Macrogen, Japan (https://www.macrogen-japan.co.jp/), for whole-genome sequencing using an Illumina platform sequencer and the TruSeq DNA PCR Free (350) Library Kit (Rhodes *et al.* 2014). The NGS raw data (FASTQ format) were then transformed and mapped to the reference rice genome Os-Nipponbare-Reference-IRGSP-1.0 pseudomolecules Release 7 (Kawahara *et al.* 2013) using the Bowtie2 v. 2.2.5 mapping tool. Finally, the UnifiedGenotyper tool in GATK v.3.8 (Depristo *et al.* 2011) was used for variant calling and to generate the VCF file for both albino and green plants. Further details are provided in Supplemental Text 1.

### Sanger sequencing for NGS data validation

To validate the observed insertion in the *swl1-R332P* gene, we amplified this locus and re-sequenced it using Sanger sequencing. The Sanger re-sequencing was done using an Applied Biosystems 3130 Genetic Analyzer (Mardis 2017, Sanger and Coulson 1975, Sanger *et al.* 1977) according to the manufacturer’s instructions. Further details are provided in Supplemental Text 1.

### Genotyping *SWL1*

To evaluate the albinism and its allelic characteristics, we genotyped 298 seeds. To specifically analyze our desired genomic region, we used a derived cleaved amplified polymorphic sequence (CAPS) technique (Neff *et al.* 1998) with the two primers SWL1-F and SWL1-R (Supplemental Table 2) that were designed using Primer3-Plus software (https://www.bioinformatics.nl/cgi-bin/primer3plus/primer3plus.cgi) and supplied by Hokkaido System Science Co., Ltd. Along with the pair of primers, we also used the restriction enzyme site‐specific endodeoxyribonuclease Cfr10 I (Takara Bio USA, Inc.) that recognizes and cleaves the sequence R/CCGGY to generate DNA fragments with 5ʹ tetranucleotide extensions (Janulaitis *et al.* 1983). The recognition sequence was present only in amplicons derived from gDNA extracted from albino plants. DNA isolation for genotyping was done using a simple DNA extraction protocol (Park *et al.* 2014) with few modifications. Further details are provided in Supplemental Text 1.

### Transmission electron microscopy

To confirm whether the albino mutants described in our study were similar to the *swl1* mutants discovered by Hayashi-Tsugane *et al.* (2014), we used the TEM protocol presented in their paper. The leaf samples were pre-fixed with 2% glutaraldehyde and 1% paraformaldehyde in 50 mM cacodylate buffer (pH 7.2) at 4°C overnight and rinsed in the same buffer. The samples were then post-fixed with 1% osmium tetroxide for 3 hours, dehydrated in a graded series of ethanol, and embedded in epoxy resin at 60°C. Ultrathin sections (70 nm) were made using an EM UC7 ultramicrotome (Leica Microsystems). Semithin sections were stained with toluidine blue and observed with a conventional light microscope. Ultrathin sections were stained with 2% uranyl acetate for 15 min, stained in a lead staining solution for 3 min, and examined using a transmission electron microscope (JEM-2100, JEOL) at 80 kV.

## Results

### A breeder seed population of ‘Hinohikari’ shows a stable albinism frequency

In 2012, breeder seed from the Hinohikari variety started to display albino mutant seedlings in the Miyakonojo field of MCAES. To investigate the albino frequency in this breeder seed population, we performed a first series of trials in 2015 in which we evaluated 5438 seeds. This screen identified 74 albino seedlings (1.361%). In the second series of trials, 2281 seeds randomly sampled from the same seed population were evaluated in 2019 at Hokkaido University. In these trials, 31 seedlings (1.359%) were albino. The third series of experiments, performed in 2020 at Hokkaido University, included 1476 randomly selected seeds from the same population. In these experiments, 20 seedlings (1.355%) were albino. Therefore, across all three experiments, a total of 9195 seeds were raised and evaluated, and the albinism incidence was maintained and stable at 1.36% (Table 1). The albino plantlets grew poorly compared to green plants and died after producing only a few leaves (Supplemental Fig. 1).

### Whole-genome NGS reveals genomic differences between green and albino plants

Because albinism frequency was stable at 1.36% for every experiment, we were able to identify the genetic cause of this phenotype as a natural mutation using NGS analysis. To do so, we treated a group of albino plants as a mutant population and a group of green plants from the same population as a non-mutant (wild type) population. The two populations were used to characterize their genomic differences. We prepared the two pools of genomic DNAs (gDNAs) by mixing 13 and 10 gDNA samples isolated from individual green and albino seedlings, respectively. To assure quality and equal representation in each pooled DNA sample, we ensured that each individual contributed the same amount of final DNA (10 ng) (Supplemental Table 1). We then deep sequenced the two gDNA pools at 15 GB. The sequencing results contained more than 30 million short reads of 150-bp-long paired ends for each pool. The quality of the two sequenced pools was almost the same in terms of total read bases, total reads, GC (%), AT (%), probability of a correct base call of 99% (Q20), and probability of a correct base call of 99.9% (Q30), with just slight non-significant differences. The Q20 sequencing quality values were 96.94% and 97.03% and the Q30 values were 92.06% and 92.31% for the green and albino pools, respectively. Total bases retained after quality trimming were 96.34% and 96.31% for green and albino pools, respectively, ensuring that a high percentage of quality reads were retained for downstream analysis (Supplemental Table 3).

### Genomic differences between green and albino plants

**Fig. 1.**
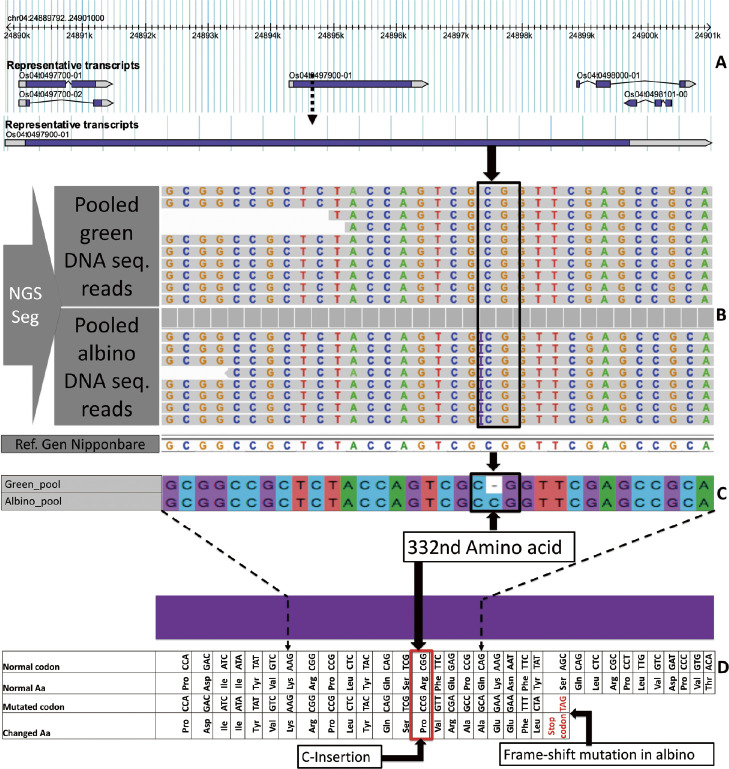
Identification of C insertion in the *SWL1* locus of albino mutants from Hinohikari breeder seed. (A) The *SWL1* locus (Os04g0497900) among other genes on Chr. 4 as extracted from the rice annotation project (RAP) database. (B) WGS-NGS analysis of the genomic differences between pooled green plants and pooled albino plants at the 24895349 position on Chr. 4, where the pooled albino sample contains the C insertion in all mapped reads. (C) Sanger sequencing results confirming C insertion in the pooled albino sample, whereas the pooled green sample had no insertion. (D) The effects of C insertion, whereby the 332nd amino acid arginine is changed to proline, ultimately resulting in a premature TAG stop codon.

Our primary objective was to find the genomic differences between green (wild type) and albino (mutant) populations. High-quality reads retained after preprocessing for green and albino gDNA pools were mapped to the Nipponbare reference genome IRGSP1.0, producing a total of 336,844 variants. Of the quality-filtered reads, 30.867 × 10^6^ (99.65%) and 30.613 × 10^6^ (99.62%) were successfully mapped in the green sample and albino sample respectively, yielding a sequencing depth of approximately 10× (Supplemental Table 3). After filtering (3 < reads numbers < 100), 158,266 polymorphic sites (46.99%) were retained for downstream analysis. As a result of the filtering, based on our rule of thumb (Supplemental Text 1), 217 of the 158,266 sites fit the criteria; 105 of these were SNPs, 40 were insertions, and 72 were deletions. Out of the 217 variants annotated, 93 (43%) were associated with protein-coding genes. Upon selection of hypothesized albinism causative genes after annotation, there were 18 genes with high, moderate, or low putative impact (Supplemental Table 4). Using the Integrated Genomic Viewer (IGV, Robinson *et al.* 2017), we found that eight of the 18 genes had their variant positions well mapped and showed clear differences between albino and green gDNA samples (Table 2). Of these eight genes, one (Os04g0497900) had a C base insertion in all mapped reads from the albino gDNA pool, but not in any mapped reads from the green gDNA pool (Fig. 1A, 1B) (Table 2). This candidate gene is referred to as *Snow-White Leaf 1* (*SWL1*) in the literature (Hayashi-Tsugane *et al.* 2014) and is described as an “unknown protein with the N-terminal chloroplast transit peptide, formation of thylakoid membranes” in RAP-DB (https://rapdb.dna.affrc.go.jp). To confirm the C insertion in albino plants using another sequencing platform, we also Sanger sequenced the *swl1* locus, which corroborated the presence of the insertion in albino plants (Fig. 1C). This C insertion is located on chromosome (Chr.) 4 between bases 24895349 and 24895350, in the *SWL1* gene and results in a change of the 332nd amino acid from arginine to proline; we designated the latter form as the *swl1-R332P* allele. The *swl1-R332P* allele leads to a truncated protein, as the resulting frameshift produces a premature stop codon (TAG) at position 24895373 on Chr. 4 (Fig. 1D). These results implied that the C insertion in *SWL1* could be the causative mutation for the albino phenotype.

### *swl1-R332P* mutation is associated with the albino phenotype

**Fig. 2.**
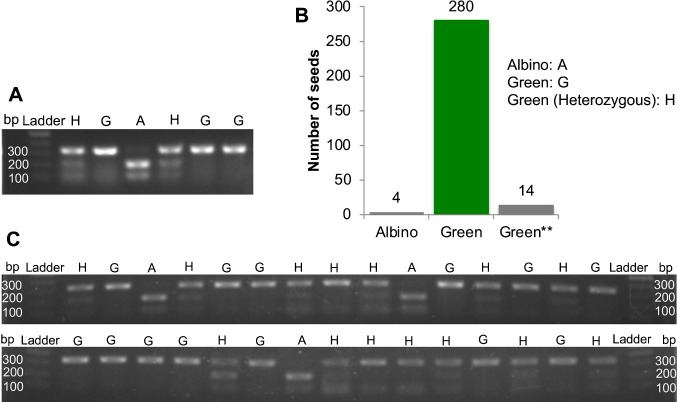
Genotype and phenotype results of 298 plants. (A) CAPS markers differentiating green (G), with a single band at 300 bp; albino (A), with double bands at 200 and 100 bp; and heterozygous (H) plants, with triple bands at 300, 200, and 100 bp. (B) Genotypes of 298 randomly selected plants from the Hinohikari breeder seed, of which 4 were homozygous albino, 14 were heterozygous green, and 280 were homozygous green. (C) Thirty of the 298 genotyped plants showing differences in band positions as presented in A.

Next, we designed DNA marker at the *SWL1* locus for genotyping with cleaved amplified polymorphic sequences (CAPSs) and the restriction enzyme Cfr10I, which cleaves the CCGG sequence present only in the albino genome. This CAPS marker was designed to distinguish the three genotypes: homozygous *swl1-R332P* (A), homozygous wild type (G), and heterozygous (H) (Fig. 2A). The electrophoretic profiles were as follows: the G genotype had a single band at 300 bp, the A genotype showed double bands at 200 and 100 bp, and the H genotype displayed three bands at 300, 200, and 100 bp (Fig. 2A, 2C). Out of 298 plants randomly selected from the Hinohikari breeder seed population, four albino plants were shown to have the A genotype, 280 green plants had the G genotype, and 14 green plants displayed the H genotype (Fig. 2B, Supplemental Table 5). Thus, the albino phenotype completely corresponded to the A genotype, whereas the green phenotype was found exclusively in plants with either the G or H genotype.

### Validation of the *swl1* phenotype

**Fig. 3.**
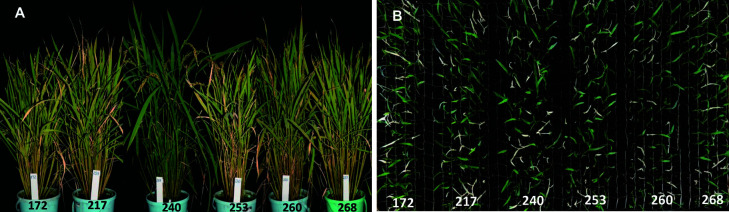
Segregation of the albino phenotype from *SWL1* heterozygous plants. (A) Six of the 14 *SWL1* heterozygous plants that were retained for self-crossing. Each plant produced thousands of seeds, from which about 100 were randomly sampled and grown to observe their segregation behavior. (B) Segregation of the heterozygous plants grown from about 100 seeds randomly sampled from the plants in A. The albino plants had white leaves, and non-albino plants had green leaves.

To confirm the relationship between genotype and phenotype, we genotyped about 100 segregant offspring seedlings descended from six of the heterozygous plants (Fig. 3). The segregant plants produced green seedlings as well as albino mutant seedlings, which grew into weak plants with a total lack of chlorophyll and poor growth compared to the green seedlings (Fig. 3, Supplemental Fig. 1). The green and albino seedlings occurred in a phenotypic ratio that did not differ significantly from the expected 3:1 for a recessive trait, as determined by a *χ*^2^ test at a cutoff of *p* ≤ 0.05 and was also confirmed by the ANOVA where there was no statistically significant difference between the observed phenotypic ratio and the expected 3:1 for a recessive trait (*p* ≤ 0.05) (Table 3, Supplemental Fig. 2). With one of the six segregant populations from the heterozygous plants, we performed genotyping at the position of the *swl1-R332P* allele, resulting in a ratio of 23 (G):51 (H):22 (A), which perfectly matched the observed phenotypic distribution (Supplemental Fig. 2). Moreover, planting 45 seeds from each of the 22 homozygous dominant plants did not result in any albino seedlings (Supplemental Table 6). Therefore, we judged that the causative mutation of the albino phenotype that occurred in Hinohikari breeder seeds coincided with homozygosity at the *swl1-R332P* allele.

### Ultrastructural leaf features of the *swl1-R332P* mutant

**Fig. 4.**
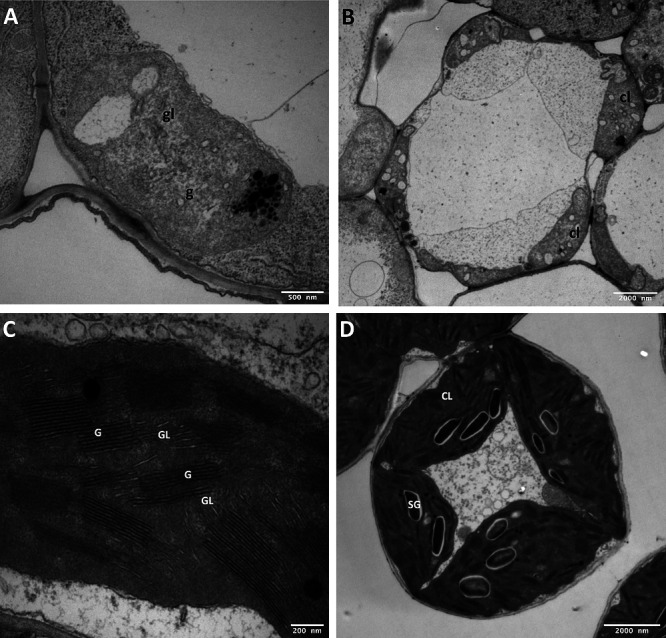
Transmission electron microscopy (TEM) pictures of the leaf ultrastructure. (A) Absence of interconnected grana and grana lamellae with no thylakoid membrane in albino leaf ultrastructure. (B) Deformed chloroplasts lacking starch granules in albino leaf ultrastructure. (C) A normal, interconnected grana and grana lamellae thylakoid membrane in green leaf ultrastructure. (D) A normal chloroplast with starch granules in green leaf ultrastructure. Abbreviations: CL: chloroplast, cl: deformed chloroplast, G: grana, g: deformed grana, GL: grana lamella, gl: deformed grana lamella, SG: starch granules. Scale bars indicate 500, 2000, 200, and 2000 nm in A, B, C, and D, respectively.

**Fig. 5.**
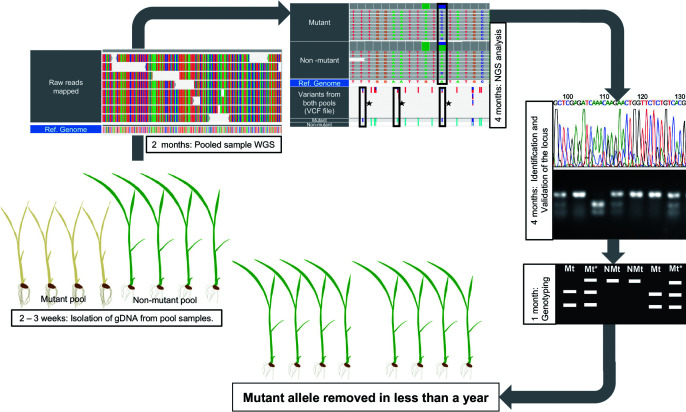
Schematic for removal of mutant alleles from seed population. First, 10–15 samples are pooled for testing. Second, the pooled sample is subjected to WGS. Third, NGS analysis is performed to extract genomic differences between the two pools. Fourth, the results are validated through processes that include re-sequencing of the segments that have differences using more accurate sequencing platforms. Fifth, allelic characteristic tests are conducted that involve self-crossing and genotyping to evaluate the nature of mutational inheritance. The possible genomic differences are Mt (mutant), NMt (non-mutant), and Mt*(mutant – heterozygous).

To examine phenotypic similarities between the *swl1-R332P* mutant and the *swl1* mutant described by Hayashi-Tsugane *et al.* (2014), we assessed leaf ultrastructure in albino and green plants. We selected albino and green plants of the same age (four-leaf stage) and sampled the third leaf from each seedling for ultrastructural transmission electron microscopy (TEM) examination. The TEM results were similar to those reported by Hayashi-Tsugane *et al.* (2014). In their findings, the *swl1* mutant leaves had deformed chloroplasts, and we also observed deformed chloroplasts in albino leaves (Fig. 4, Supplemental Fig. 2). They also found that the deformed chloroplasts lacked thylakoid membranes, as did we, with the albino leaf structures we observed having no intertwined stroma-grana thylakoid network (Fig. 4A, 4B). In contrast to the leaves of the albino *swl1* and *swl1-R332P* mutant plants, the leaves of green plants had normal chloroplast and stroma-grana thylakoid networks (Fig. 4C, 4D). We observed additional aberrant phenotypes in the albino plants, including deformed chloroplasts lacking starch, deformed mitochondria, and a deformed mesophyll (Fig. 4A, 4B, Supplemental Fig. 3A, 3B). The green leaves, however, had normal organelles, including well-structured mitochondria with observable cristae (Fig. 4C, 5D, Supplemental Fig. 3C, 3D). Thus, the albino trait was consistently associated with chloroplast abnormalities not seen in the green plants.

## Discussion

### Recessive mutation at the *SWL1* locus in the breeder seed population

In this study, we confirmed that the mutated *swl1* gene is responsible for albinism in the ‘Hinohikari’ rice variety that was bred more than 40 years ago (Yagi *et al.* 1990). The occurrence of albinism was stable in the population and followed simple Mendelian inheritance. The albino phenotype was 1.36% in the breeder seed population (Table 1). However, the mutant allele in this population should be present at more than 1.36% in this breeder seeds population because its allele was latent as heterozygous. The frequency of this mutant allele may change every generation. The albino homozygous allele is lethal and does not inherit, although the mutation allele transmits to next generation as a heterozygous form (Hoffman *et al.* 2004) and can be difficult to identify (Williams 1999). Recessive alleles can remain latent in the population in the form of heterozygotes. The heterozygous and homozygous dominant individuals were phenotypically identical (Supplemental Fig. 1). Because recessive mutations are not readily apparent, they are difficult to remove quickly or completely (Hoffman *et al.* 2004), as the recessive phenotype appears only after homozygotes are produced through reproduction (Allard and Hansche 1964). Albinism caused by the recessive *swl1-R332P* allele (Table 3) will be more difficult to remove from the population compared to a dominant mutation. To satisfy DUS criteria for a variety, a precise and rapid method needs to be developed to remove similar deleterious mutations. We propose here a whole-genome sequencing (WGS) approach using NGS analysis to identify the causative gene responsible for a mutation, as represented in Fig. 5. The results from our analyses can be used to direct breeders in removing unwanted mutations. In our case, non-mutant Hinohikari seeds can be selected only from plants homozygous for the dominant *SWL1* allele, which do not produce albino plants in the next generation.

### Identification of mutated allele using NGS leads to rapid elimination of it from the breeder seed population

Before NGS, map-based cloning analysis required at least 3 years to identify a gene of interest, even for mutants showing simple Mendelian inheritance with a single gene mutation (Jander *et al.* 2002). A conventional method may also take at least 3 years to remove a mutation from a contaminated population even after it is identified: a year to collect seed from plants showing non-mutant phenotypes, a second year to select plants that do not segregate the mutant phenotype, and a third year to reconfirm non-segregant plants. Moreover, it is difficult to detect mutations caused by SNPs or small indels in a population with the same genetic background using conventional DNA markers such as SSR, as these markers require more than a single base pair of repeating DNA sequences (Kage *et al.* 2016). Therefore, the conventional screening method was less time-consuming, less expensive, and more feasible for identifying mutant genes and removing individuals with mutant alleles from breeder seeds. Although DNA-based technologies including gene isolation have assisted in promoting crop breeding programs (Fang *et al.* 2016), the purpose of gene isolation by researchers and the use of the isolated genes have been considerably disconnected in practice from maintenance of the breeder seeds. However, if the mutant and normal populations are simply analyzed at the WGS level, the gene can be easily identified by searching for genomic sites that show 100% linkage to the mutant trait. As this case study shows, it took about 1 year to employ NGS analysis to find the target gene, and then the mutated allele was rapidly removed. So, identifying mutant genes in breeder seeds, which used to be carried out using advanced molecular genetic methods, can now be performed using the simple process of comparing whole-genome sequences. If the genes responsible for the mutations can be easily found, they can be used for the actual management of breeder seeds (Rasmussen *et al.* 2010). Identification of the mutated gene enables rapid and precise removal of the mutant allele. This is a major advantage for institutions that control the propagation and quality of breeder seeds. Supplying healthy seeds is important for gaining the confidence of growers and consumers.

### Genomic variants detected in the breeder seed population

We expected to find a single genomic difference between green and albino plants, as albinism might be controlled by a mutation or polymorphism at a single locus. Surprisingly, we detected 217 polymorphisms upon comparing green and albino plants, including the C insertion on the *SWL1* gene that causes albinism. This points to the fact that, apart from the albino mutation, there are 216 additional genomic differences in the population. The percentage of reads linked with phenotypes for the 217 variants (with exception of the *SWL1* locus) was below 100 (Table 2, Supplemental Table 4), perhaps due to accumulation of *SWL1*-unrelated mutations in the population. The 216 variant genetic differences detected in this study are considered mutations that did not affect phenotype. At present, we do not know whether these variants are genetic polymorphisms or errors derived from the NGS analysis. A comparative study done in *Arabidopsis thaliana* for a parent-progeny relationship revealed a higher mutation frequency in heterozygotes, but only a few mutations involved essential genes (Yang *et al.* 2015). However, the Hinohikari population may contain mutations that have accumulated over the 40 years during which it has been bred. Many old varieties may have undergone a similar accumulation of spontaneous mutations due to SNPs (Hofmeister *et al.* 2020), indels (Dubrovina and Kiselev 2016), and transposable elements (Quesneville 2020). Potentially serious mutations that could affect useful traits in subsequent generations might arise through these processes. It is highly likely that the *swl1-R332P* mutation in this case was caused by the manifestation of a latent accumulated mutation. It may be possible to predict the frequency and nature of spontaneously occurring mutations in breeder seeds by examining how the 216 variant polymorphisms found in this NGS data.

### For protecting breeder seeds from undesired mutations

Maintenance of cultivars is best accomplished by ensuring that there is enough breeder seed stored at optimal conditions for many years; if that is not possible, the next best option is progeny selection, in which the breeding stock is selected based on the performance of its offspring or descendants (Parlevliet 2007). To protect breeder seeds from mutations, progeny selection can also be performed by growing a small population, with the first step being to divide the population into smaller populations and growing them separately (Ikeda *et al.* 2007). The second step is that when a deleterious phenotype is found in a small population, all seeds from that population are then excluded from distribution and proliferation. Seeds from the previous population may also not be used because such deleterious variations might be heterozygous and must have formed in the previous generation in order to be revealed in the next generation (Kono *et al.* 2018). The third step would be for the seed producer to proliferate seeds from separate populations that have not developed mutations. To appropriately manage the breeder seeds, it is important to divide the entire group of seeds into several lines with the scale corresponding to a total amount that is enough to supply to farmers.

In this research, we have designed an analysis method coupling WGS with NGS to rapidly remove deleterious mutations from a population to maintain breeder seed quality. A spontaneous mutation in breeder seeds that leads to a mutant phenotype can easily be detected by the method presented here. This should prove useful to institutions responsible for keeping, proliferating, maintaining, and distributing seeds. The mutations’ potential to affect the seed population can thus be minimized because it will take only a single year to completely remove such mutations from the seed population. In the case of the albino mutation, the deleterious phenotype was controlled by a recessive allele and was detectable only when in a homozygous state. We were able to completely remove the deleterious mutation after identifying the *swl1-R332P* allele to obtain the healthy Hinohikari seeds. However, we detected another 216 variant genomic polymorphisms in the breeder seed population, which may imply that the accumulation of mutations could result in unforeseen variation in future generations.

## Author Contribution Statement

EGB and YKi planed research. EGB, MSD and YKi designed research. YN prepared rice breeder seed samples. EGB carried out experiments. EGB, MSD, SS, and KY analyzed data. EGB and TI observed ultrastructure of leaf cells. SS, KY, YKa, and YKo assisted ddRAD seq analysis. EGB and YKi wrote and improved manuscript. YKo and YKi supervised EGB’s PhD study.

## Supplementary Material

Supplemental Figures

Supplemental Tables

Supplemental Text

## Figures and Tables

**Table 1.** Occurrence of albino of the Hinohikari breeder’s seed in MCAES*^a^*

Materials tested	Number of albino occurrences (counts)	Total numbers of seeds tested	Incidence (%)	Year & place of testing
Original population	74	5438	1.361	2015 at MCAES
Testing 1	31	2281	1.359	2019 at Hokkaido University
Testing 2	20	1476	1.355	2020 at Hokkaido University
Total	125	9195	1.359	

*^a^* MCAES: Miyazaki Comprehensive Agricultural Experiment Station, Japan.

**Table 2.** Eight most suspected genes causing albinism

Gene involved	Polymorphism		Position of polymorphism from the start codon	% reads linked with phenotypes*^a^*	Description*^b^*
	Green	Albino			
Os04g0497900	GC	GCC	992	100.00	Unknown protein with the N-terminal chloroplast transit peptide, Formation of thylakoid membranes
Os04g0367000	A	C	4103	74.07	Hypothetical conserved gene
Os02g0558200	A	G	1736	72.41	Hypothetical protein
Os05g0427800	A	G	80	70.73	Similar to Ribulose bisphosphate carboxylase large chain
Os01g0936800	G	C	9446	68.18	PapD-like domain containing protein
Os09g0120800	T	C	2139	67.86	Similar to ATPase, calcium-transporting-related (Fragment)
Os05g0431333	G	A	in 5ʹUTR	64.52	Hypothetical gene
Os11g0655000	AGAT	AGATGAT	3699	64.29	Conserved hypothetical protein

*^a^* % reads linked with phenotypes is calculated by taking percentage of sum of green wild type reads and albino mutated reads divide by sum of green wild type reads, green mutated reads, albino wild type reads and albino mutated reads. *^b^* Gene descriptions are obtained from the rice annotation project database website: RAP-DP at https://rapdb.dna.affrc.go.jp/.

**Table 3.** Segregation of progeny from six *SWL1* heterozygous lines *χ*^2^ test and ANOVA test

Heterozygous seed	Total seedlings	Green	Albino	*χ*^2^ test				ANOVA test	
				Chi-Square *^a^*	*p*-value	Remarks		Pr(>F)^b^	Remarks
172	79	60	19	0.038	0.8 < *p* < 0.9	NS		0.07283	NS
217	92	70	22	0.058	0.8 < *p* < 0.9	NS		0.07086	NS
240	94	70	24	0.014	0.8 < *p* < 0.9	NS		0.0735	NS
253	104	77	27	0.051	0.8 < *p* < 0.9	NS		0.07653	NS
260	79	58	21	0.105	0.7 < *p* < 0.8	NS		0.06274	NS
268	91	70	21	0.179	0.5 < *p* < 0.7	NS		0.06014	NS

*^a^* Any lines were not significantly different at the *p* = 0.05 level for an expected ratio of the 3:1 segregation when *χ*^2^ test was conducted. *^b^* Any lines were not significantly different at the *p* = 0.05 level for an expected Mendelian ratio of the 3:1 segregation when ANOVA test was conducted. NS: not significant.
