# Supplementary material for: Seed management using NGS technology to rapidly eliminate a deleterious allele from rice breeder seeds
Source: Breed Sci. 2022 Dec 13;72(5):362–71. doi: 10.1270/jsbbs.22058 (PMC9895803; doi:10.1270/jsbbs.22058)
Supplement: Supplementary file 1 — Supplemental Figures [file 72_362_s1.pdf]

doi: 10.1270/jsbbs.22058

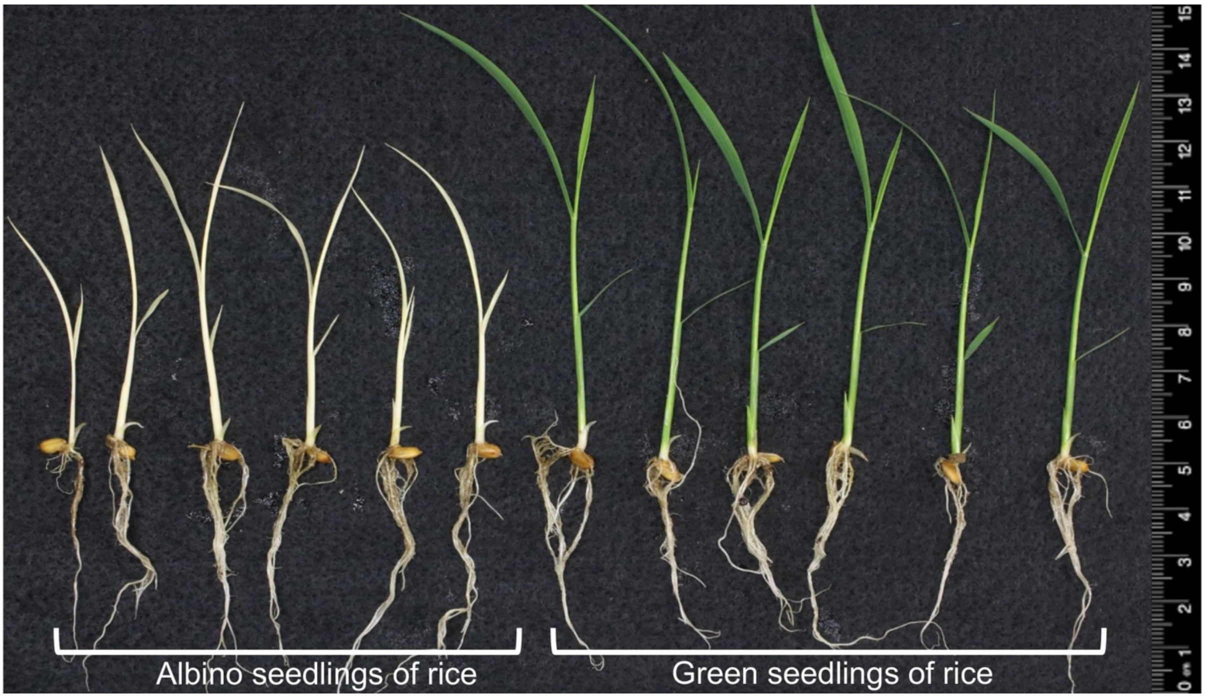

Supplemental Fig. 1: Growth efficiency comparison between albino and green plants.
At the same age (three-leaf seedlings 3 weeks after planting), green seedlings are bigger than the albino seedlings, and albino seedlings eventually die.

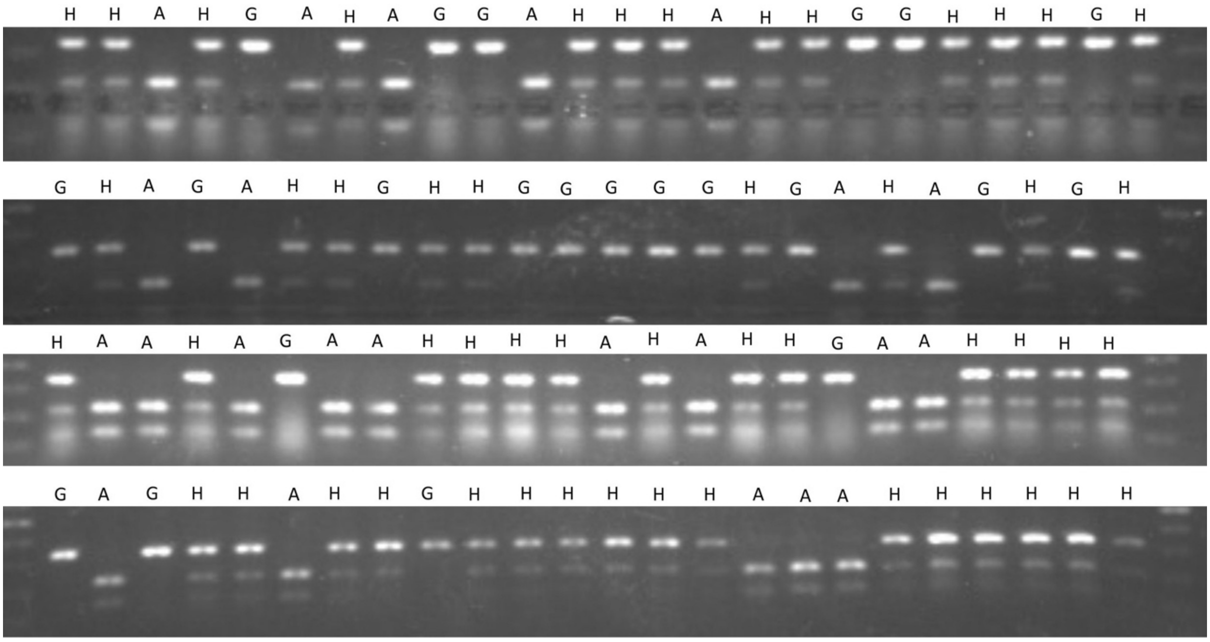

Supplemental Fig. 2: Genotyping results of 96 F2 plants from F1 *SWL1* heterozygote.
G, H, and A represent green, heterozygous, and albino, respectively. Results were 23G:51H:22A with a χ^2 value of 0.105485232 and *p*-value of 0.75, which is not significantly different from the expected Mendelian ratio for a recessive mutation, supporting the characterization of the *swl1-332* mutation as recessive.

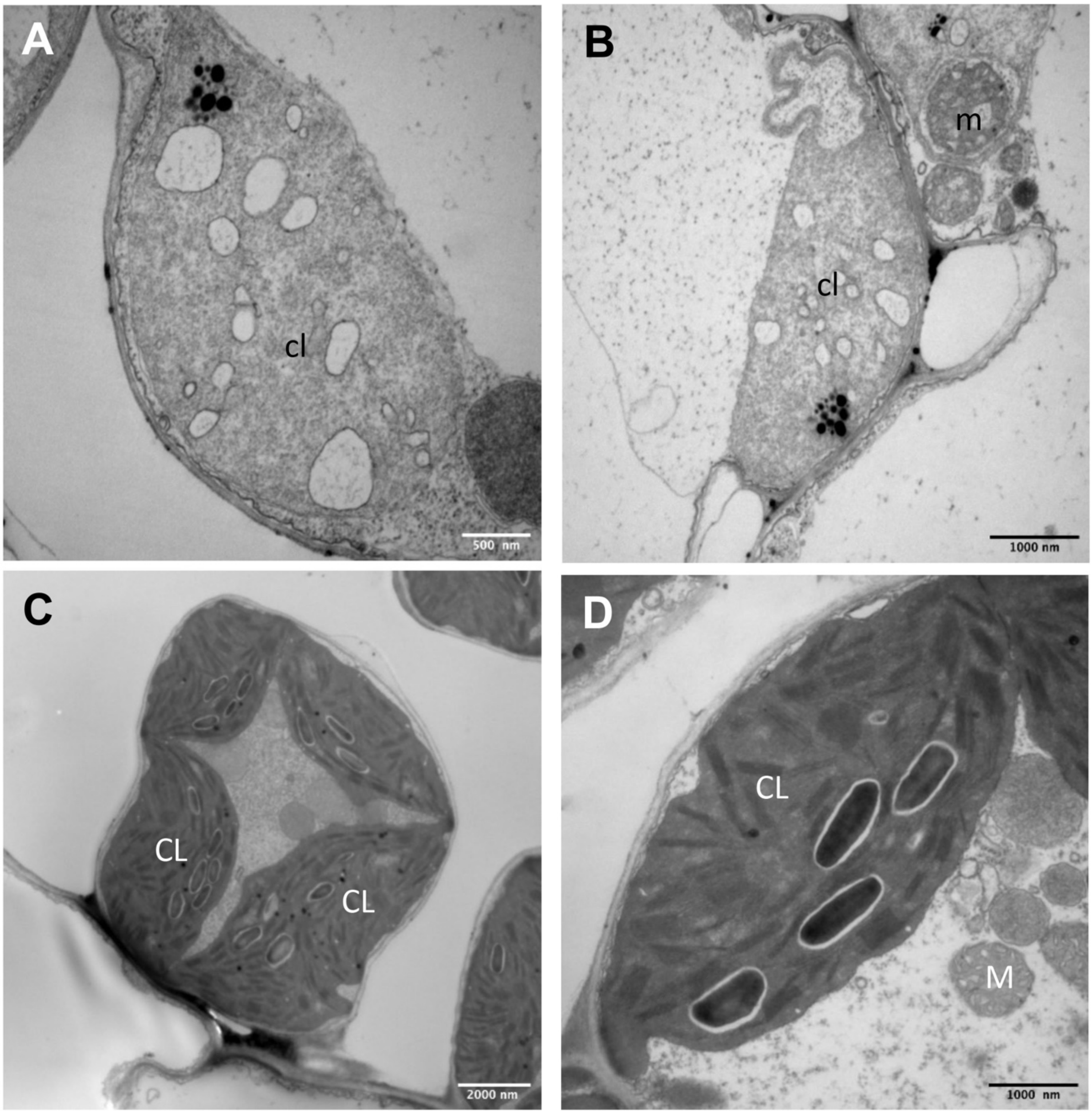

Supplemental Fig. 3: Transmission Electron Microscope (TEM) pictures of the leaf ultrastructure. (A) deformed chloroplast with no starch granules in albino leaf ultrastructure. (B) deformed chloroplasts with no mitochondria in albino leaf ultrastructure. (C) a well chloroplast structure in green leaf ultrastructure. (D) chloroplast with starch granules and mitochondria in green leaf ultrastructure. Each abbreviation is as follows; CL: chloroplast, M: mitochondria, cl: deformed chloroplast, m: deformed mitochondria. Scale bar indicates 500nm, 1000nm, 2000nm and 1000nm in lengths for A, B, C, and D respectively.
