# Supplementary material for: Seed management using NGS technology to rapidly eliminate a deleterious allele from rice breeder seeds
Source: Breed Sci. 2022 Dec 13;72(5):362–71. doi: 10.1270/jsbbs.22058 (PMC9895803; doi:10.1270/jsbbs.22058)
Supplement: Supplementary file 2 — Supplemental Tables [file 72_362_s2.pdf]

doi: 10.1270/jsbbs.22058

Supplemental Table 1: Pooled albino and green samples, with individual gDNA concentrations.

S/N	Sample name	Concentration (ng/uL)	260/280	Volume (uL)
		**Albino samples**		
1	AL07	45.9	1.92	10.9
2	AL09	52.9	1.88	9
3	AL10	45.9	1.91	10.9
4	AL11	54.7	1.88	8.4
5	AL12	38.4	1.87	9.1
6	AL13	21.7	1.97	10
7	AL15	40.2	1.86	10.5
8	AL17	62.4	1.97	8
9	AL20	111.1	1.87	7
10	AL25	73.5	1.83	7.3
**Total to make 10ng from each sample**				**91.1**
		**Green samples**		
1	GR04	74.5	1.88	6.7
2	GR05	41.1	1.89	6.1
3	GR06	64.6	1.88	7.7
4	GR08	35.3	1.82	9.3
5	GR09	68.2	1.92	7.3
6	GR10	93.3	1.9	6
7	GR11	69.7	1.92	7.2
8	GR12	56	1.89	8.9
9	GR13	80.2	1.91	6.2
10	GR14	82.7	1.88	6
11	GR15	50.8	1.86	9.8
12	GR16	90.3	1.89	6
13	GR19	85.9	1.88	5.8
**Total to make 10ng from each sample**				**93.2**

The abbreviations AL and GR represent albino and green samples, respectively. The numbers after AL or GR relate to sample numbers. All numbers are in sequence; for missing samples, the gDNA did not attain the quality cutoffs of an A_{260}/A_{280} ratio of at least 1.8 and a concentration of at least 20 ng/μL. The volume (μL) of each sample corresponds to the total volume of each sample that contributed 10 ng of each sample to the pool.

Supplemental Table 2: Primers and restriction enzyme used for genotyping to detect the *swl1-332* allele.

**Product name**	**Type**	**Sequence**
SWL1 (Os04g0497900)-F	Primer - F	GGCTTGACAAATGGAACCTC
SWL1 (Os04g0497900)-R	Primer - R	AGGCACCTGTTTTTGTCCAC
Cfr10 I	Restriction Enzyme	G/CCGGT

Both primers were used to amplify the forward and reverse direction of the C insertion for PCR reactions, and the same forward primer was used to bind the nucleotides on single-stranded DNA in Sanger sequencing.

Supplemental Table 3: Quality attributes of sequence data.

Quality attribute	Samples: Pooled green	Samples: Pooled albino
Reads # (Million)	30.977 (99.63 %)	30.729 (99.61 %)
Total bases retained after trimming (Billion)	4.516 (96.34 %)	4.479 (96.31 %)
Mean Reads Length	145.8	145.77
Paired Reads after trimming (Million)	30.867 (99.65 %)	30.613 (99.62 %)
Paired total bases (Billion)	4.500 (99.65 %)	4.462 (99.62 %)
Unpaired Reads #:	109676 (0.35 %)	116768 (0.38 %)
Unpaired total bases (Million):	15.989 (0.35 %)	17.021 (0.38 %)
Discarded reads #	113832 (0.37 %)	121144 (0.39 %)
Trimmed bases (Million):	171.669 (3.66 %)	171.444 (3.69 %)
Reads Filtered by length cutoff (30 bp):	2973 (0.01 %)	3652 (0.01 %)
Bases Filtered by length cutoff:	42993 (0.00 %)	51436 (0.00 %)
Reads Filtered by continuous base "N" (3)	0 (0.00 %)	0 (0.00 %)
Bases Filtered by continuous base "N"	0 (0.00 %)	0 (0.00 %)
Reads Filtered by low complexity ratio (0.8):	47016 (0.15 %)	50287 (0.16 %)
Bases Filtered by low complexity ratio (Million)	6.863 (0.15%)	7.341 (0.16%)
Reads Filtered by avg quality (20.0):	63843 (0.21 %)	67205 (0.22 %)
Bases Filtered by avg quality (Million)	9.313 (0.20 %)	9.804 (0.21 %)
Reads Trimmed by quality (5.0)	0 (0.00 %)	2 (0.00 %)
Bases Trimmed by quality	0 (0.00 %)	2 (0.00 %)
GC (%)	43.61%	43.98%
AT (%)	56.39%	56.02%
Q20 (%)	96.94%	97.03%
Q30 (%)	92.06%	92.31%

Q20 means that the sequencing quality is 99% accurate, allowing only 1/100 probability of incorrectness, and Q30 means that the sequencing quality is 99.9% accurate, allowing only 1/1000 probability of incorrectness.

Supplemental Table 4: Eighteen candidate genes that could cause albinism

S/N	Gene involved	Position	Polymorphism: Green	Polymorphism: Albino	Polymorphism: Putative impact	Green: Wild type reads	Green: Mutated reads	Albino: Wild type reads	Albino: Mutated reads	% Reads linked with phenotypes	Descrition
1	Os04g0497900	24895349	GC	GCC	High	14	0	0	8	100.00	Unknown protein with the N-terminal chloroplast transit peptide, Formation of thylakoid membranes
2	Os04g0367000	17765453	A	C	High	11	6	1	9	74.07	Hypothetical conserved gene
3	Os02g0558200	21152820	A	G	High	12	6	2	9	72.41	Hypothetical protein
4	Os02g0558100	21157702	T	G	High	12	6	2	8	71.43	Chloride channel (Fragment)
5	Os05g0427800	20973964	T	G	Low	8	6	6	21	70.73	Similar to Ribulose bisphosphate carboxylase large chain
6	Os01g0936900	41126873	G	C	Low	7	3	4	8	68.18	Peptidase A1 domain containing protein
7	Os09g0120800	1615064	A	C	Low	8	7	2	11	67.86	Similar to ATPase, calcium-transporting-related (Fragment)
8	Os05g0431333	21165114	G	A	Low	11	9	2	9	64.52	Hypothetical gene
9	Os11g0655000	26207406	AGAT	AGATGAT	Low	13	0	10	5	64.29	Conserved hypothetical protein
10	Os02g0558100	21152819	A	G	Low	12	6	2	2	63.64	Similar to Chloride channel (Fragment)
11	Os04g0367900	17868697	T	G	Moderate	11	7	4	8	63.33	Similar to H0607F01.2 protein
12	Os11g0620500	24193216	CC	C	Moderate	12	3	6	2	60.87	Tyrosine protein kinase domain containing protein
13	Os12g0208900	5676270	AA	A	Moderate	13	5	6	4	60.71	Similar to ZF-HD protein dimerisation region containing protein, expressed
14	Os02g0528900	19405572	T	G	Moderate	6	6	2	6	60.00	Similar to PDR-like ABC transporter
15	Os04g0367800	17868690	G	C	Moderate	11	7	4	5	59.26	Conserved hypothetical protein
16	Os01g0936800	41126873	G	C	Moderate	7	3	4	3	58.82	PapD-like domain containing protein
17	Os04g0370900	18041892	T	A	Moderate	7	7	3	7	58.33	Similar to cDNA, clone: J013067H13, full insert sequence
18	Os04g0370900	18041899	G	T	Moderate	7	7	3	4	52.38	Similar to H0607F01.5 protein

The % reads linked with phenotypes is calculated by taking a percentage of the sum of green wild-type reads and albino mutated reads divided by the sum of green wild-type reads, green mutated reads, albino wild-type reads, and albino mutated reads. The gene description was obtained from the rice annotation project database website. Putative impact is equivalent to deleterious effects.

Supplemental Table 5: Genotyping results of 298 lines for SWL1 gene

S/N	Ind #	Score	S/N	Ind #	Score	S/N	Ind #	Score	S/N	Ind #	Score
1	MS1	G	45	MS46	G	89	MS90	G	133	MS134	G
2	MS2	G	46	MS47	G	90	MS91	G	134	MS135	G
3	MS3	G	47	MS48	G	91	MS92	G	135	MS136	G
4	MS4	G	48	MS49	G	92	MS93	G	136	MS137	G
5	MS5	G	49	MS50	G	93	MS94	G	137	MS138	G
6	MS6	G	50	MS51	G**	94	MS95	G	138	MS139	G
7	MS7	G	51	MS52	G	95	MS96	A	139	MS140	G
8	MS8	G	52	MS53	G	96	MS97	G	140	MS141	G
9	MS9	G	53	MS54	G	97	MS98	G	141	MS142	G
10	MS10	G	54	MS55	G	98	MS99	G	142	MS143	G
11	MS11	G	55	MS56	G	99	MS100	G	143	MS144	G
12	MS12	G	56	MS57	G	100	MS101	G	144	MS145	G
13	MS13	G	57	MS58	G	101	MS102	G	145	MS146	G
14	MS14	G**	58	MS59	G	102	MS103	A	146	MS147	G
15	MS15	G	59	MS60	G	103	MS104	G	147	MS148	G
16	MS16	G	60	MS61	G	104	MS105	G	148	MS149	G
17	MS17	G	61	MS62	G	105	MS106	G	149	MS150	G
18	MS18	G	62	MS63	G	106	MS107	G	150	MS151	G
19	MS19	G	63	MS64	G	107	MS108	G	151	MS152	G
20	MS20	G	64	MS65	G	108	MS109	G	152	MS153	G
21	MS21	G	65	MS66	G	109	MS110	G	153	MS154	G
22	MS22	G**	66	MS67	G	110	MS111	G	154	MS155	G
23	MS23	G	67	MS68	G	111	MS112	G	155	MS156	G
24	MS24	G	68	MS69	G	112	MS113	G	156	MS157	G
25	MS26	G	69	MS70	G	113	MS114	G	157	MS158	G
26	MS27	G	70	MS71	G**	114	MS115	G	158	MS159	G
27	MS28	G	71	MS72	G	115	MS116	G	159	MS160	G
28	MS29	G	72	MS73	G	116	MS117	G	160	MS161	G
29	MS30	A	73	MS74	G	117	MS118	G	161	MS162	G
30	MS31	G	74	MS75	G	118	MS119	G	162	MS163	G
31	MS32	G	75	MS76	G	119	MS120	G	163	MS164	G
32	MS33	G	76	MS77	G	120	MS121	G	164	MS165	G
33	MS34	G	77	MS78	G**	121	MS122	G	165	MS166	G
34	MS35	G	78	MS79	G	122	MS123	G	166	MS167	G
35	MS36	G	79	MS80	G	123	MS124	G	167	MS168	G
36	MS37	G	80	MS81	G	124	MS125	G	168	MS169	G
37	MS38	G	81	MS82	G	125	MS126	G	169	MS170	G
38	MS39	G	82	MS83	G	126	MS127	G	170	MS171	G
39	MS40	G	83	MS84	G	127	MS128	G	171	MS172	G**
40	MS41	G	84	MS85	G	128	MS129	G	172	MS173	G
41	MS42	G	85	MS86	G	129	MS130	G	173	MS174	G
42	MS43	G	86	MS87	G	130	MS131	G	174	MS175	G
43	MS44	G	87	MS88	G	131	MS132	G	175	MS176	G
44	MS45	G	88	MS89	G	132	MS133	G	176	MS177	G

Notes: A: Homozygus Albino, G: Homozygous Green and G**: Heterozygous

Supplemental Table 5 continues: Genotyping results of 298 lines for SWL1 gene

S/N	Ind #	Score	S/N	Ind #	Score	S/N	Ind #	Score
177	MS178	G	219	MS221	G	261	MS263	G
178	MS179	G	220	MS222	G	262	MS264	G
179	MS180	G	221	MS223	G	263	MS265	G
180	MS181	G	222	MS224	G	264	MS266	G
181	MS182	G	223	MS225	G	265	MS267	G
182	MS183	G	224	MS226	G	266	MS268	G**
183	MS184	G	225	MS227	G	267	MS269	G
184	MS185	G	226	MS228	G	268	MS270	G
185	MS186	G	227	MS229	G	269	MS271	G
186	MS187	G	228	MS230	G	270	MS272	G
187	MS188	G	229	MS231	G**	271	MS273	G
188	MS189	G	230	MS232	G	272	MS274	G
189	MS190	G	231	MS233	G	273	MS275	G
190	MS191	G	232	MS234	G	274	MS276	G**
191	MS192	G	233	MS235	G	275	MS277	G
192	MS193	G	234	MS236	G	276	MS278	G
193	MS194	G	235	MS237	G	277	MS279	G
194	MS195	G	236	MS238	G	278	MS280	G
195	MS196	G	237	MS239	G	279	MS281	G
196	MS197	G	238	MS240	G**	280	MS282	G
197	MS198	G	239	MS241	G	281	MS283	G
198	MS200	G	240	MS242	G	282	MS284	G
199	MS201	G	241	MS243	G	283	MS285	G
200	MS202	G**	242	MS244	G	284	MS286	G
201	MS203	G	243	MS245	G	285	MS287	G
202	MS204	G	244	MS246	G	286	MS288	G
203	MS205	G	245	MS247	G	287	MS289	G
204	MS206	G	246	MS248	G	288	MS290	G
205	MS207	G	247	MS249	G	289	MS291	G
206	MS208	G	248	MS250	G	290	MS292	G
207	MS209	G	249	MS251	G	291	MS293	G
208	MS210	G	250	MS252	G	292	MS294	A
209	MS211	G	251	MS253	G**	293	MS295	G
210	MS212	G	252	MS254	G	294	MS296	G
211	MS213	G	253	MS255	G	295	MS297	G
212	MS214	G	254	MS256	G	296	MS298	G
213	MS215	G	255	MS257	G	297	MS299	G
214	MS216	G	256	MS258	G	298	MS300	G
215	MS217	G**	257	MS259	G			
216	MS218	G	258	MS260	G			
217	MS219	G	259	MS261	G			
218	MS220	G	260	MS262	G			

Notes: A: Homozygus Albino, G: Homozygous Green and G**: Heterozygous

Supplemental Table 6: Testing of plants homozygous for the dominant *swl1-332* locus

Plant ID	No_seeds tested	Replication	Albino Frequency	Green Frequency
MS1	20	I	0	20
MS8	20	I	0	20
MS18	20	I	0	20
MS35	20	I	0	20
MS41	20	I	0	20
MS46	20	I	0	20
MS53	20	I	0	20
MS63	20	I	0	20
MS79	20	I	0	20
MS85	20	I	0	20
MS90	20	I	0	20
MS97	20	I	0	20
MS107	20	I	0	20
MS123	20	I	0	20
MS129	20	I	0	20
MS134	20	I	0	20
MS141	20	I	0	20
MS151	20	I	0	20
MS158	20	I	0	20
MS160	20	I	0	20
MS167	20	I	0	20
MS173	20	I	0	20
MS1	25	II	0	20
MS8	25	II	0	20
MS18	25	II	0	20
MS35	25	II	0	20
MS41	25	II	0	20
MS46	25	II	0	20
MS53	25	II	0	20
MS63	25	II	0	20
MS79	25	II	0	20
MS85	25	II	0	20
MS90	25	II	0	20
MS97	25	II	0	20
MS107	25	II	0	20
MS123	25	II	0	20
MS129	25	II	0	20
MS134	25	II	0	20
MS141	25	II	0	20
MS151	25	II	0	20
MS158	25	II	0	20
MS160	25	II	0	20
MS167	25	II	0	20
MS173	25	II	0	20

The abbreviation MS followed by numbers refers to Miyazaki Seeds.
