# Supplementary material for: Seed management using NGS technology to rapidly eliminate a deleterious allele from rice breeder seeds
Source: Breed Sci. 2022 Dec 13;72(5):362–71. doi: 10.1270/jsbbs.22058 (PMC9895803; doi:10.1270/jsbbs.22058)
Supplement: Supplementary file 3 — Supplemental Text [file 72_362_s3.pdf]

doi: 10.1270/jsbbs.22058

Supplemental Text 1.

Materials and Methods

Next generation sequencing and Analysis

The NGS raw data (FASTQ) were trimmed by first removing adapters using cutadapt v. 1.15 (Martin 2011) and then filtered using FastQ Quality Control Software (FaQCs) (Lo and Chain 2014, O'Halloran 2017) to retain only reads of at least Q20 quality and minimum read length of 30 bp. This data manipulation was essential to have a good proportional of uniquely mapped reads for better downstream analyses. Then the filtered NGS data of the two genomic sequences (pooled green and pooled albino) were mapped to the reference rice genome Os-Nipponbare-Reference-IRGSP-1.0 pseudomolecules Release 7 (Kawahara *et al*. 2013) using a mapping tool bowtie2 v. 2.2.5 (Langmead and Salzberg 2012). To fix the read-mate pair information, we used a FixMateInformation tool in Picard v. 2.20.8 (Broad Institute 2018, Landman and Hwang 2017). MarkDuplicates also from Picard v. 2.20.8 was used to locate and tags duplicate reads (Broad Institute 2018, Zhao 2018). RealignerTargetCreator and IndelRealigner tools in The Genome Analysis Toolkit (GATK) v. 3.8 were used to identify InDels and realign reads around InDels (Depristo *et al*. 2011, McKenna *et al*. 2010). Finally, the UnifiedGenotyper tool from GATK v.3.8 (Depristo *et al*. 2011) was used for variant calling (SNP and InDel) of both albino and green samples. We then extracted all of the variants from their corresponding binary alignment and map (BAM) file so that we could verify and filter them using their mapping qualities.

Python utility pysamstats v1.1.2 (Miles 2013) was used for computational statistics against genome positions based on sequence alignments from BAM files of pooled green and albino. We decided to set 10 folds (100 reads) of the average reads to be an upper limit reads per loci and at least 3 reads lower limit. So, sites that had >100 reads and <3 reads were trashed out to minimize type I error and type II error that may arise due high sequencing coverage of the pooled samples (Cutler and Jensen 2010). The variant filtering based on a rule of thumb that "The first important variants for albinism are those with *mismatch indices of between 0.5 and 1 for albino while 0 for green, and the second important variants for albinism are those with mismatch indices of between 0.5 and 0.9 for both albino and green. All variants with **insertions or deletion indices of between 0.1 and 1 at albino while 0 at green may cause albinism". We assume from 0.5 to 1 because under pooled sample, it is possible for the loci to have minor and major alleles (https://doi.org/10.48550/arXiv.1604.04735) but we care for those of >=50%. All the assumptions help to identify the most possible causative mutation of the trait. We annotated the obtained variants using snpEff annotation tool (Cingolani *et al*. 2012) with the built-in annotation database *Oryza sativa* from a gene transfer format (GTF) file of ensemble plants release 46 (Bolser *et al*. 2017).

Sanger sequencing for NGS data validation

To validate the insertion on the SWL1 gene, we amplified the genome segment with C-insertion on SWL1 gene and accurately sequence it using sanger sequencing. The sanger re-sequencing was done by applied biosystems 3130 genetic analyzer (Mardis 2017, Sanger and Coulson 1975, Sanger *et al*. 1977) according to manufactures' instructions. Using Primer3-Plus software (http://www.bioinformatics.nl/cgi-bin/primer3plus/primer3plus.cgi) we designed two primers *SWL1* (Os04t0497900)-F and *SWL1* (Os04t0497900)-R to amplify the suspected albinism site of gDNA in both green and albino. The parameters for primer design included 200–400 bp amplicon size, 18 - 20bp optimum primer size, 50°C–60°C primer melting temperature (Tm) and 50%–60% primer GC content. The same forward primer *SWL1* (Os04t0497900)-F was also used during PCR for single-strand DNA synthesis (**Supplemental Table 2**).

Sanger-sequencing procedures

Amplification of the genome segment with C-insertion

The 50μL PCR reaction was used (0.25μL Ex-Taq, 5μL 10x sequence buffer, 4μL dNTPs, 2μL forward primers, 2μL reverse primer, 4μL template DNA and 32.75μL water). The PCR was run at 94°C for 30 sec, 39 circles of 94°C for 30 sec, 56°C for 30 sec and 72°C for 30 sec; then followed 72°C for 1 minute and 10°C at infinity. Using 5μL of PCR products with 1% gel on 1X TAE buffer, 0.5μL 10x loading buffer, 1μL midolin green, and 1.5μL 100bp marker running for 30 minutes at 100V, we identified only one band for both sample DNA. This result prompted us to purify the DNA direct from the PCR product

using FastGene Gel/PCR Extraction Kit (**NIPPON Genetics Europe**). 40µL PCR product was added into 200µL Buffer GP1 and voltexed, the sample was then loaded onto the column and centrifuged for 30 seconds at 13,000rpm. For membrane washing, the pass through was discarded and 600µL of GP2 was added into same column and centrifuged for 30 seconds at 13,000rpm and discard the pass through. For membrane drying, the column with sample was centrifuged for 2 minutes at 13,000rpm and for final Elution 30µL of GP3 was added and let it elute for 2 minutes under room temperature and then was centrifuged for 2 minutes at 13,000 rpm. At all stages, a new tube was used with the same column.

The PCR for single-strand DNA synthesis

20µL PCR reaction was used (4µL of 5x sequencing buffer, 0.32µL of forward primer, 1µL purified polymerized template DNA, 0.68µL pre mix, and 14µL water). Then PCR was run for 96°C for 30 sec, 25 circles (96°C for 10 sec and 56°C for 5 sec), 60°C for 4 min finally 10°C infinity. After PCR reaction, 2µL 3M Sodium acetate (NaOAc) and 50µL 100%ethanol were added. The sample was further purified for sequencing reaction by ethanol settling where PCR sample was transferred to 1.5mL tube and incubated for 20 min at room temperature, it was then centrifuged for 20 min at 13,000rpm, the supernatant was discarded by syringe, 100µL 70% ethanol was added and centrifuged for 10 min at 13,000rpm and discarded the supernatant by syringe, vacuum drying was done for 5 minutes and then 10µL Hi-Di was added and voltexed and finally the sample was transferred to 0.2mL tube, the sample was kept at 95°C for 2 min by thermal cycler then quick cooling on ice for 5 min was done. The resequencing was done by applied biosystems 3130 genetic analyzer (Mardis 2017) according to manufactures' instructions. Sanger re-sequenced results were viewed by MEGA X v. 10.2.4 (Kumar *et al.* 2018, Stecher *et al.* 2020).

SWL1 genotyping

DNA isolation for genotying was done using simple DNA extraction protocal (Park *et al.* 2014) with little modifications. About 2g leaf sample with two metal beads were placed in 2.0mL sampling tube, solidified with liquid nitrogen for one minute and grind with µT-01 for 30 second at 1000rpm. 350µL KCl extraction buffer was added, voltex for 2 minutes and incubated at room temperature for 5 Minutes. It was voltexed again for 2 minutes and centrifuged for 20 minutes at 13000 rpm under room temperature. The 250 - 300µL supernatant was collected into a new 1.5mL tube, 250µL of phenol chloroform added, voltexed for 2 minutes, and centrifuged for 5 minutes at 13000 rpm under room temperature. The 200 - 250µL supernatant was collected into a new 1.5mL tube, 200µL of chloroform added, voltexed for 2 minutes, and centrifuged for 5 minutes at 13000 rpm under room temperature. The 150 - 200µL supernatant was collected into a new 1.5mL tube, 150µL of 2-propanol (isopropanol) added, mixed by inversion, and centrifuge at 14100 rpm for 10 minutes under room temperature. The supernatant was discarded keeping the pellet, 200µL of 70% ethanol was added and mix by inversion then centrifuged at 13000rpm for 5 minutes at 4°C. The ethanol was discarded, and the DNA pellet was dried in a desiccator under reduced pressure for 5 minutes. Lastly the pellet was eluted by 50µL TE buffer.

For gel electrophoresis, we had a reaction mixture of 2 µL template DNA, 3 µL of H_2O, 1.25 µL of forward primer, 1.25 µL of reverse primer and 7.5 µL Quick Taq polymerase .

The PCR for genotyping was done using 10µL PCR reaction (1µL template DNA, 2µL water, 1µL reverse primer, 1µL forward primer, 5µL Quick Taq polymerase) and the PCR protocol was 94°C for 2 minutes, 32 circles of 94°C for 30 sec, 56°C for 30 sec and 72°C for 30 sec; then 10°C at infinity. After PCR, the Cfr10 I enzyme treatment was done by adding 0.02µL Cfr10 I enzyme and 1µL of 10x Cfr10 I loading buffer and incubate for 7 hours at 37 °C to make sure that the enzymes digests the entire template DNA. The gel electrophoresis was done using 1% agarose in TBE buffer, running for 30 minutes at 100V.

*Mismatch index (indices) is the measure of polymorphism calculated by taking the ratio of the number of reads whose nucleotides do not match the nucleotide of the reference sequence at a position divide by the total number of reads at that same position. For instance, the "no polymorphism" where all the reads have nucleotides like that of reference sequence has a mismatch index of 0, while the 100% polymorphism where all reads of the alternated sequence have nucleotides different from the reference sequence has a mismatch index of 1.

**Insertion or deletion index (indices) is the measure of insertions or deletions calculated by taking the ratio of the number of reads with insertions or deletions at a position divide by the total number of reads at that same position. For instance, the "no insertion or deletion" where all reads have no insertion nor

deletion has an insertion or deletion index of 0, while the 100% insertion or deletion where all reads have insertions or deletions has an insertion or deletion index of 1.

Literature Cited

Bolser, D. M., D. M. Staines, E. Perry and P. J. Kersey (2017). Ensembl plants: Integrating tools for visualizing, mining, and analyzing plant genomic data. In J. M. Walker (Ed.) Methods mol biol 1533: 1–31.

Broad Institute. (2018). *Picard Tools - GitHub*. Release 2.20.8. https://github.com/broadinstitute/picard/

Cingolani, P., A. Platts, L. L. Wang, M. Coon, T. Nguyen, L. Wang, S. J. Land, X. Lu and D. M. Ruden (2012) A program for annotating and predicting the effects of single nucleotide polymorphisms, SnpEff: SNPs in the genome of Drosophila melanogaster strain w1118; iso-2; iso-3. Fly 6: 80–92.

Cutler, D. J and J. D. Jensen (2010) To pool, or not to pool? Genetics 186: 41–43.

Kumar, S., G. Stecher, M. Li, C. Knyaz and K. Tamura (2018) MEGA X: Molecular evolutionary genetics analysis across computing platforms. Mol Biol Evol 35: 1547–1549.

Landman, S. R. and T. H. Hwang (2017). Bioinformatics data analysis of next-generation sequencing data from heterogeneous tumor samples. Methods mol biol 1633: 185–192.

Langmead, B. and S. L. Salzberg (2012) Fast gapped-read alignment with Bowtie 2. Nat Methods 9: 357–359.

Lo, C. C. and P. S. G. Chain (2014) Rapid evaluation and quality control of next generation sequencing data with FaQCs. BMC Bioinformatics 15: 1–8.

Martin, M. (2011) Cutadapt removes adapter sequences from high-throughput sequencing reads. EMBnet J 17: 10–12.

McKenna, A., M. Hanna, E. Banks, A. Sivachenko, K. Cibulskis, A. Kernytsky, K. Garimella, D. Altshuler, S. Gabriel, M. Daly *et al*. (2010) The genome analysis toolkit: A MapReduce framework for analyzing next-generation DNA sequencing data. Genome Res 20: 1297–1303.

Miles, A. (2013). pysamstats: a fast Python and command-line utility for extracting simple statistics against genome positions based on sequence alignments from a SAM or BAM file. https://github.com/alimanfoo/%0Apysamstats.

O'Halloran, D. M (2017) FastQ-brew: Module for analysis, preprocessing, and reformatting of FASTQ sequence data. BMC Res Notes 10: 1–4.

Stecher, G., K. Tamura and S. Kumar (2020). Molecular evolutionary genetics analysis (MEGA) for macOS. Mol Biol Evol 37: 1237–1239.

Zhao, Q. (2018) A study on optimizing markduplicate in genome sequencing pipeline. In Proceedings of the 2018 5th International Conference on Bioinformatics Research and Applications ACM Int. Conf. Proceeding Ser, Association for Computing Machinery, New York, pp. 8–15.
